# Periprocedural Results and Right Ventricular Outcomes of Computer Assisted Vacuum Thrombectomy Treatment of Acute Pulmonary Embolism: Interim Analysis of 300 Patients From the STRIKE‐PE Study

**DOI:** 10.1161/JAHA.124.039975

**Published:** 2025-08-29

**Authors:** John M. Moriarty, Brian J. Schiro, Suhail Y. Dohad, Houman Tamaddon, Hugh C. Davis, David M. Shavelle, George S. Chrysant, Elias A. Iliadis, David J. Dexter, Juan J. Ciampi Dopazo, Andrew Holden, Frances M. West, Brent Keeling, Andrew S. P. Sharp, Ido Weinberg

**Affiliations:** ^1^ UCLA Health Los Angeles CA USA; ^2^ Miami Cardiac & Vascular Institute Miami FL USA; ^3^ Cedars‐Sinai Medical Center Los Angeles CA USA; ^4^ Piedmont Augusta Augusta GA USA; ^5^ University of Florida – Shands Hospital Gainesville FL USA; ^6^ MemorialCare Heart & Vascular Institute Long Beach CA USA; ^7^ INTEGRIS Baptist Medical Center / INTEGRIS Cardiovascular Physicians Oklahoma City OK USA; ^8^ Cooper University Hospital Camden NJ USA; ^9^ Sentara Vascular Specialists Norfolk VA USA; ^10^ Hospital Universitario Virgen de las Nieves in Granada Granada Spain; ^11^ Auckland University School of Medicine Auckland New Zealand; ^12^ Thomas Jefferson University Hospital Philadelphia PA USA; ^13^ Emory University Hospital Midtown Atlanta GA USA; ^14^ University College Dublin Dublin Ireland; ^15^ Hadassah Hebrew University Medical Center Jerusalem Israel

**Keywords:** Computer assisted vacuum thrombectomy, lung embolism, mechanical thrombectomy, prospective studies, pulmonary embolism, Clinical Studies, Hemodynamics, Echocardiography, Treatment, Embolism

## Abstract

**Background:**

Pulmonary embolism (PE) can be a life‐threatening condition. Endovascular treatment is emerging as a promising treatment to restore hemodynamic stability and reverse right ventricular (RV) dysfunction in PE; however, more studies are needed to elucidate the effects on the right ventricle after endovascular treatment. This analysis reports the effects of computer assisted vacuum thrombectomy on RV function.

**Methods:**

Eligible patients for the single‐arm, prospective, international, multicenter STRIKE‐PE (A Prospective, Multicenter Study of the Indigo Aspiration System Seeking to Evaluate the Long‐Term Safety and Outcomes of Treating Pulmonary Embolism) cohort study are adults with acute PE with symptoms for ≤14 days and an RV/left ventricular ratio of ≥0.9 who are treated with computer assisted vacuum thrombectomy. Reported here are periprocedural and RV outcomes of the initial 300 patients of STRIKE‐PE, to provide insights into effects on RV function after computer assisted vacuum thrombectomy.

**Results:**

Patients were classified with high‐risk (5.7%), intermediate‐high‐risk (84.7%), or intermediate‐low‐risk (9.7%) PE. Median thrombectomy time was 30 minutes. Mean on‐table systolic pulmonary artery pressure decreased from 51.7 mm Hg to 41.3 mm Hg, a 19.1% reduction (*P*<0.001). The change in mean RV/left ventricular ratio from baseline to 48 hours postprocedure (primary effectiveness end point) was a decrease from 1.40 to 0.99, a 26.8% reduction (*P*<0.001). Clinical parameters and echocardiographic measures of right heart strain improved from baseline to 48 hours postprocedure (*P*<0.001). The rate of composite major adverse events within 48 hours postprocedure (primary safety end point) was 2.0%. Median Borg dyspnea scale at rest decreased from 4.0 at baseline to 0.5 at discharge (*P*<0.001).

**Conclusions:**

This interim analysis demonstrates that computer assisted vacuum thrombectomy safely and expeditiously improves hemodynamic status, RV function, and perceived dyspnea.

**Registration: URL:**

https://clinicaltrials.gov; Unique Identifier: NCT04798261.

## Graphical abstract

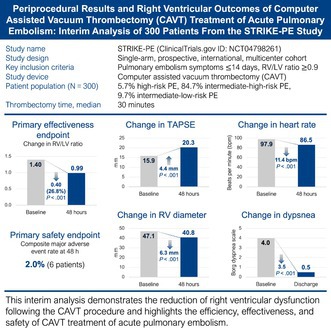

**Nonstandard Abbreviations and Acronyms**
CAVT: computer assisted vacuum thrombectomy
EVT: endovascular treatment
MAEs: major adverse events
TAPSE: tricuspid annular plane systolic excursion

**Clinical Perspective**

**What Is New?**

- This is the largest cohort to date of patients with pulmonary embolism undergoing computer assisted vacuum thrombectomy—a mechanical thrombectomy system with a computer algorithm to regulate aspiration; this study describes favorable periprocedural changes in hemodynamic status, right ventricular function, and perceived dyspnea.

**What Are the Clinical Implications?**

- The improvement in right ventricular function suggests that computer assisted vacuum thrombectomy may reduce metrics of excessive right ventricular afterload.
- These findings underscore the safety and effectiveness of computer assisted vacuum thrombectomy as a treatment option for intermediate‐risk and high‐risk pulmonary embolism.

Pulmonary embolism (PE) is the third leading cause of cardiovascular death, behind myocardial infarction and stroke.^1^ Early recognition and management of PE are crucial to reduce morbidity and mortality, particularly for patients classified as high‐risk or intermediate‐risk PE per European Society of Cardiology guidelines.^2^ Goals of treatment options in these 2 classifications are to prevent mortality or clinical deterioration via focusing on hemodynamic stability and right ventricular (RV) function.^2^, ^3^, ^4^

In the past decade, endovascular treatment (EVT) of acute PE has become a promising therapy to quickly improve hemodynamics and RV function.^5^, ^6^ Current guidelines suggest the use of EVT in high‐risk PE as a bailout option after failure of systemic reperfusion or in patients with high bleeding risk; limited evidence exists for guidelines to recommend the use of EVT in intermediate‐risk PE.^2^, ^3^, ^4^, ^7^, ^8^ Furthermore, these guidelines do not factor in additional variables that may increase the use of EVT.

A variety of mechanical thrombectomy and catheter‐directed thrombolysis devices for EVT of acute PE has been studied.^9^, ^10^ In contrast to catheter‐directed thrombolysis, mechanical thrombectomy directly and immediately removes thrombus and does not require a thrombolytic drug. Current clinical evidence demonstrates that mechanical thrombectomy is a safe and effective treatment of acute PE.^11^, ^12^, ^13^, ^14^, ^15^ More specifically, an early study reports that computer assisted vacuum thrombectomy (CAVT) is safe and effective for treating intermediate‐risk and high‐risk PE.^12^ More clinical data are needed to strengthen these findings in patients with intermediate‐risk or high‐risk PE.

The STRIKE‐PE (A Prospective, Multicenter Study of the Indigo Aspiration System Seeking to Evaluate the Long‐Term Safety and Outcomes of Treating Pulmonary Embolism) study is designed to evaluate safety, effectiveness, and functional outcomes and quality of life of an intermediate‐risk and high‐risk acute PE population treated with CAVT using the Indigo Aspiration System (Penumbra Inc). This study will enroll up to 1500 patients internationally and is the largest study to date on CAVT using the Indigo Aspiration System. This article reports the interim analysis of periprocedural outcomes, hemodynamic status, and RV function of the first 300 consecutive patients in this study.

## Methods

### Study Design

The clinical trial is registered with https://www.clinicaltrials.gov as NCT04798261. The data that support the findings of this study may be made available from the corresponding authors upon reasonable request.

**Figure 1:**
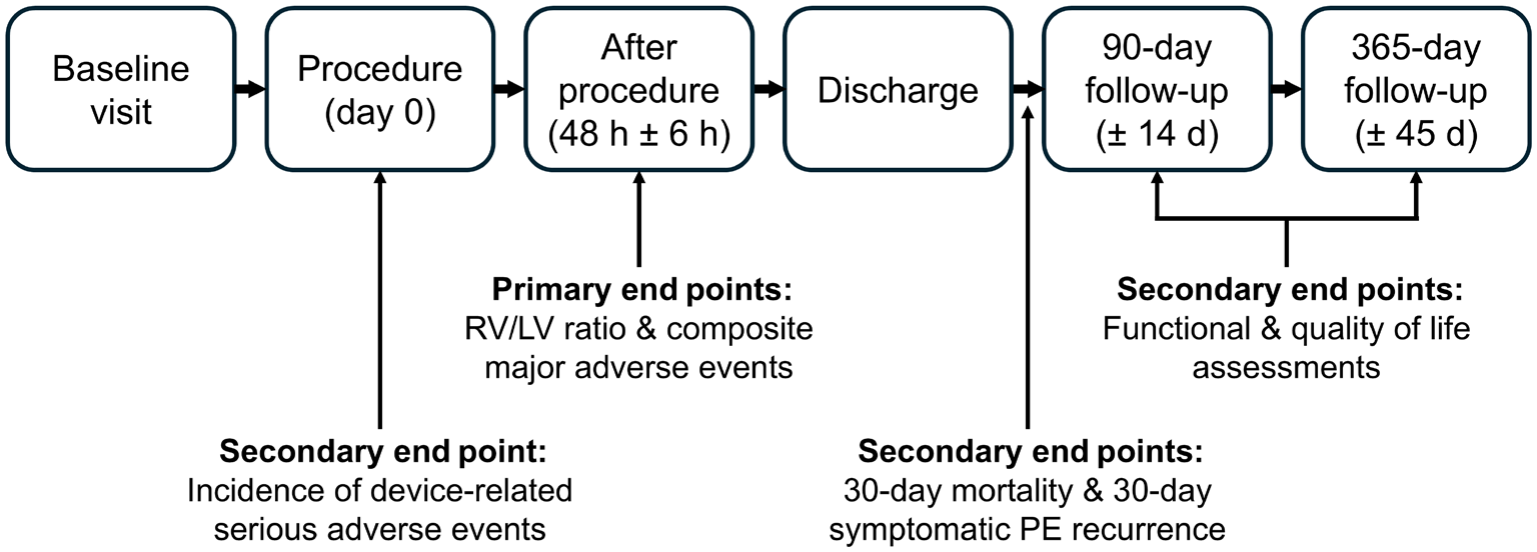
Overview of STRIKE‐PE study design with primary and secondary end points at each follow‐up period. PE indicates pulmonary embolism; RV/LV, right ventricular/left ventricular; and STRIKE‐PE, A Prospective, Multicenter Study of the Indigo Aspiration System Seeking to Evaluate the Long‐Term Safety and Outcomes of Treating Pulmonary Embolism.

The study design for the single‐arm, prospective, international, multicenter STRIKE‐PE cohort study is summarized here and reported in detail elsewhere.^12^ Eligible patients are adults with acute PE with symptoms for ≤14 days and an RV/left ventricular (RV/LV) ratio of ≥0.9 who receive treatment with the Indigo Aspiration System and from whom informed consent is obtained. Data are collected for up to 1 year (Figure 1). Reported in this interim analysis are periprocedural (to 30 days) data from the first 300 enrolled patients, treated from June 2021 through October 2023 by 89 physicians across 37 sites (31 in the United States and 6 in Europe; additional site principal investigators and treating physicians of this STRIKE‐PE analysis are listed in Table S1). Before enrolling patients, each site obtained institutional review board/ethics committee approval and completed site‐specific requirements, and all enrolled patients provided informed consent.

### Devices

Patients are treated with mechanical thrombectomy with an algorithm device, referred to as CAVT (Indigo Aspiration System, Penumbra Inc), with the goal of removing thrombus in a single treatment session. The systems used in this STRIKE‐PE analysis include 12F (Lightning 12) and 16F (Lightning Flash) aspiration catheters and computer assisted Lightning tubing that regulates aspiration by using a microprocessor, pressure sensors, flow sensors (for 16F aspiration catheters), and high‐frequency electromechanical valves. The 16F aspiration catheters became commercially available in the United States after being Food and Drug Administration approved in early 2023 and they received Conformité Européenne mark in September 2024.

### Data Collected

Patient population and presentation, clinical procedural characteristics, and effectiveness and safety data were collected as previously reported.^12^ The primary effectiveness end point for the STRIKE‐PE study is the change in RV/LV ratio from baseline to 48 hours postprocedure, calculated by using matched imaging pairs. The RV/LV ratio was determined by using either echocardiography or computed tomography pulmonary angiography on the basis of the availability of the imaging modality at the site of the treating physician. When RV/LV ratio data were available from both imaging modalities, computed tomography pulmonary angiography was the preferred modality. For sites that elected to participate in having the core laboratory evaluate the RV/LV ratio, data from the core laboratory superseded physician‐reported data. The primary safety end point is a composite of major adverse events (MAEs; device‐related death, major bleeding, device‐related clinical deterioration, device‐related pulmonary vascular injury, and device‐related cardiac injury) at 48 hours postprocedure.^16^, ^17^ The secondary safety end points for the STRIKE‐PE study are the incidences of device‐related serious adverse events, all‐cause mortality within 30 days, and symptomatic PE recurrence within 30 days. All these safety end points were adjudicated by an independent medical reviewer.

Additionally, clinical parameters and echocardiography measures of right heart strain were collected. Heart rate, respiratory rate, the number of patients on supplemental O_2_, and the supplemental O_2_ amount were measured at baseline and at 48 hours postprocedure. Collection of echocardiography images was encouraged but was at the discretion of the physicians.

Echocardiography images acquired at baseline and at 48 hours postprocedure were evaluated. A core laboratory measured tricuspid annular plane systolic excursion (TAPSE) and identified the presence of McConnell's sign—impaired radial contraction with preserved apical long axis contraction. If both the physician and the core laboratory measured RV diameter, the core laboratory data superseded the physician data.

### Statistical Analysis

All statistical analyses were performed on the intention‐to‐treat population, defined as all enrolled patients. Data were analyzed as available; missing data were not imputed. Patients were either admitted into the intensive care unit (ICU) after the procedure or not. For the patients who were admitted into the ICU, the median ICU stay was calculated from only those patients.

The numbers and percentages of patients who achieved clinically relevant thresholds for modified Miller score (a measure of clot burden on a computed tomography pulmonary angiography scan) at presentation and for TAPSE and RV diameter at baseline and at 48 hours postprocedure were calculated. A modified Miller score of ≥12 correlates with increased RV strain, pulmonary artery diameter, and likelihood of septal shift.^18^ A TAPSE of <16 mm correlates with an unfavorable prognosis.^19^ An RV diameter of >42 mm indicates dilatation of the right ventricle.^20^

Descriptive statistics were calculated and reported as mean±SD, median (Q_1_–Q_3_), or count (percentage). The 2‐tailed paired *t* test, Wilcoxon signed rank test, or McNemar test were performed as appropriate to calculate significance of differences in paired data. All statistical analyses were performed by using SAS software (version 9.4, SAS Institute).

## Results

### Patient Baseline Characteristics and Presentation

**Figure 2:**
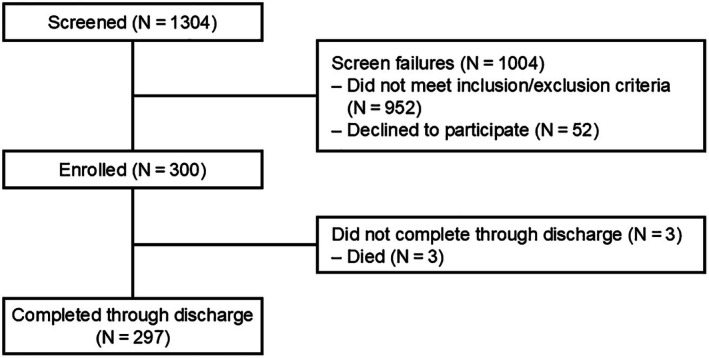
Flow diagram for this analysis (N=300) of the STRIKE‐PE study to discharge. STRIKE‐PE indicates A Prospective, Multicenter Study of the Indigo Aspiration System Seeking to Evaluate the Long‐Term Safety and Outcomes of Treating Pulmonary Embolism.

For these 300 patients (Table 1; Table S2; flow diagram in Figure 2), mean age was 61.2±14.8 years and 161 (53.7%) were men. Notable medical history included deep vein thrombosis (45.3%), previous PE (11.7%), and history of cancer (12.0%). Median time from symptom onset to admission was 41.0 hours (14.0–104.5). Seventeen (5.7%) patients were classified with high‐risk PE, 254 (84.7%) with intermediate‐high‐risk PE, and 29 (9.7%) with intermediate‐low‐risk PE.^7^ A simplified pulmonary embolism severity index of ≥1 was present in 245 (81.7%) patients.

**Table 1:** Patient Baseline Characteristics and Presentation

Demographics	No.=300
Age, y	61.2±14.8
Sex	
Men	161 (53.7%)
Women	139 (46.3%)
Race*	
American Indian or Alaska Native	1 (0.4%)
Asian	0 (0%)
Black	69 (26.2%)
White	166 (63.1%)
Other†	3 (1.1%)
Unknown or not reported	24 (9.1%)
Ethnicity*	
Hispanic or Latino	32 (12.2%)
Not Hispanic or Latino	226 (85.9%)
Unknown or not reported	5 (1.9%)
Body mass index, kg/m^2^	34.2±8.6
Comorbidities	
Hypertension	170 (56.7%)
Deep vein thrombosis	136 (45.3%)
Hyperlipidemia	119 (39.7%)
Diabetes	74 (24.7%)
COVID‐19 (active)	11 (3.7%)
History of cancer	36 (12.0%)
Previous pulmonary embolism	35 (11.7%)
Time from symptom onset to admission,‡ h	41.0 [14.0–104.5]
Classification of pulmonary embolism early mortality risk§	
High	17 (5.7%)
Intermediate high	254 (84.7%)
Intermediate low	29 (9.7%)
Simplified pulmonary embolism severity index ≥1	245 (81.7%)
Modified Miller score	16.0 [14.0–16.0]
Modified Miller score ≥12	250 (83.3%)
Tissue plasminogen activator for the index pulmonary embolism within the past 14 d (excluding the 24 h before procedure)	0.7% (2/300)
Concomitant medications (multiple medications allowed)	
Heparin (unfractionated or low molecular weight)	300 (100%)
Other anticoagulants	10 (3.3%)
Antiplatelet agents	51 (17.0%)

Data are presented as mean±SD, median [Q_1_–Q_3_], or n (%).

* Collected only for patients in the United States (n=263).

^†^ Patients had the option to self‐report their race via free‐text entry. Two patients self‐identified as European and one patient self‐identified as “Other”.

^‡^ Patients admitted before symptom onset had time assigned as 0 h.

^§^ Per 2019 European Society of Cardiology guidelines.^7^

Median modified Miller score at presentation was 16 (14–16), and 250 (83.3%) patients had an modified Miller score of ≥12. Two (0.7%) patients received tissue plasminogen activator between 14 days and 24 hours before the procedure (administered before transfer to the treating facility), whereas all patients were receiving parenteral anticoagulation with either unfractionated or low molecular weight heparin.

### Periprocedural Characteristics and Hospital Stays

Median thrombectomy time (first CAVT device insertion to last CAVT device removal) was 30 minutes (21–44), and median procedure time (venous puncture to access site closure) was 62 minutes (48–86; Table 2). Mean estimated blood loss was 292.3±162.0 mL. Treatment was bilateral in 244 patients (81.3%; Table S3).

**Table 2:** Clinical Periprocedural Characteristics and Results of 300 Patients

Time from symptom onset to puncture, h	69.5 [35.0–127.0]
Time from admission to puncture, h	18.0 [8.0–28.0]
Thrombectomy time,* min	30.0 [21.0–44.0]†
Procedure time,‡ min	62.0 [48.0–86.0]§
Intracardiac thrombus noted	3 (1.1%)||
Intracardiac thrombus removed by CAVT devices	2 (66.7%)
Estimated blood loss, mL	292.3±162.0
On‐table pre‐CAVT systolic pulmonary artery pressure (n=294)	
<30 mm Hg	8 (2.7%)
≥30 mm Hg and ≤50 mm Hg	138 (46.9%)
>50 mm Hg and ≤70 mm Hg	123 (41.8%)
>70 mm Hg	25 (8.5%)
Lengths of stay	
ICU LOS after procedure, d	
Only patients admitted to ICU#	2.0 [1.0–3.0]
Included all patients**	1.0 [0.0–2.0]
ICU LOS after procedure, nights	
Only patients admitted to ICU#	1.0 [0.0–2.0]
Included all patients**	0.0 [0.0–1.0]
Not admitted to ICU after procedure	121 (40.3%)
Hospital LOS, d	5.0 [4.0–7.0]

Data are presented as mean±SD, median [Q_1_–Q_3_], or n (%).

CAVT indicates computer assisted vacuum thrombectomy; ICU, intensive care unit; and LOS, length of stay.

* Defined as time from first CAVT device insertion to last CAVT device removal.

^†^ n=291.

^‡^ Defined as time from venous puncture to access site closure.

^§^ n=297.

^||^ n=285.

^#^ n=179.

** Patients who did not require an ICU stay had time assigned as 0 days and nights.

Median postprocedure ICU stay was 2 days (1–3) when patients not admitted to the ICU were excluded and was 1 day (0–2) when patients not admitted to the ICU were included; 121 patients (40.3%) did not require an ICU stay after the procedure. Median hospital length of stay was 5 days (4–7).

### On‐Table Hemodynamic Outcomes

During the procedure, mean systolic pulmonary artery pressure decreased significantly from 51.7±14.5 mm Hg to 41.3±14.0 mm Hg, a reduction of 19.1%±19.7% (*P*<0.001; Table 3). Similarly, mean pulmonary artery pressure decreased significantly from 31.0±9.2 mm Hg to 24.5±8.9 mm Hg, a reduction of 20.0%±24.5% (*P*<0.001).

**Table 3:** On‐Table Hemodynamic Outcomes Before and After CAVT

Pulmonary artery pressure parameter (paired data)	Pre‐CAVT, mm Hg	Post‐CAVT, mm Hg	Absolute reduction, mm Hg	*P* value	Percent reduction, %	*P* value
Systolic pulmonary artery pressure*	51.7±14.5	41.3±14.0	10.4±10.2	<0.001	19.1±19.7	<0.001
Mean pulmonary artery pressure†	31.0±9.2	24.5±8.9	6.5±6.4	<0.001	20.0±24.5	<0.001

Data are presented as mean±SD. The paired *t* test was performed on paired mean differences.

CAVT indicates computer assisted vacuum thrombectomy; post‐CAVT, after CAVT procedure on‐table; and pre‐CAVT, before CAVT procedure on‐table.

* n=281.

^†^ n=272.

### Primary End Points: RV/LV Ratio and Composite MAEs

The change in mean RV/LV ratio from baseline to 48 hours postprocedure (primary effectiveness end point) was a decrease from 1.40±0.36 to 0.99±0.22, a reduction of 26.8%±14.9% (*P*<0.001; Table 4).

**Table 4:** Primary Safety and Effectiveness Outcomes

Primary effectiveness end point (right ventricular/left ventricular ratio, paired data)*	
Composite computed tomography pulmonary angiography and echocardiography pairs (n=285)	
Baseline	1.40±0.36
At 48 h postprocedure†	0.99±0.22
Reduction	0.40±0.30; *P*<0.001
Percent reduction, %	26.8±14.9; *P*<0.001
Primary safety end point (major adverse event within 48 h postprocedure)‡	
Composite	6 (2.0%)§
Device‐related death||	0 (0%)
Major bleeding#	6 (2.0%)**
Device‐related clinical deterioration||,, ††	2 (0.7%)
Device‐related cardiac injury||	0 (0%)
Device‐related pulmonary vascular injury|| ^y^	1 (0.3%)

Data are presented as mean±SD or n (%). The paired *t* test was performed on paired mean differences.

* All imaging modality pair classifications (computed tomography pulmonary angiography or echocardiography and evaluated by core laboratory or physician) are listed in Table S4.

^†^ Time from procedure to 48‐hour scan is a median of 44.5 hours (Q_1_–Q_3_, 41.1–48.1; n=284).

^‡^ Per independent medical reviewer.

^§^ Four access site hematomas (major bleeding) related to procedure; 1 pulmonary artery perforation (device‐related pulmonary vascular injury) leading to major bleeding and device‐related clinical deterioration; 1 pulmonary artery hemorrhage (major bleeding) leading to device‐related clinical deterioration.

^||^ Adverse events that were judged as probably or definitely related to the Indigo Aspiration System were considered to be device related.

^#^ Major bleeding is defined per Mehran et al.^16^

** Three patients (1.0%) experienced major bleeding requiring transfusion (defined as events that are independent medical reviewer–adjudicated as major bleeding within 48 hours and had action taken of transfusion); for 1 of those patients (0.3%), the transfusion was also considered device related (defined as events that are independent medical reviewer–adjudicated as probably or definitely device related and had action taken of transfusion).

^††^ Clinical deterioration is defined per Meyer et al.^17^

The composite MAE rate (primary safety end point) was 2.0% (6 patients; Table 4, Table S5). The MAEs experienced were 4 access site hematomas, 1 pulmonary artery perforation, and 1 pulmonary artery hemorrhage. Three patients (1.0%) experienced major bleeding requiring transfusion; for 1 of those patients (0.3%), the transfusion was also considered device related. No device‐related deaths or device‐related cardiac injuries occurred within 48 hours.

### Additional RV Measures and Vital Signs

From baseline to 48 hours postprocedure, the echocardiography measures of right heart strain, TAPSE, and RV diameter improved significantly (*P*<0.001) and the proportions of patients with TAPSE <16 mm, RV diameter >42 mm, and McConnell's sign decreased significantly (*P*<0.001; Table 5).

**Table 5:** Changes in Echocardiographic Measures of Right Heart Strain and Vital Signs From Baseline to 48 Hours

Parameters	Baseline	48 h postprocedure	Reduction from baseline to 48 h	*P* value
Echocardiographic measures (paired data)				
TAPSE, mm*	15.9±5.4	20.3±4.7	−4.4±4.2†	< 0.001
Right ventricular diameter, mm‡	47.1±7.4	40.8±6.1	6.3±7.3	< 0.001
TAPSE <16 mm*	49 (53.3%)	16 (17.4%)	33 (35.9%)	< 0.001
Right ventricular diameter >42 mm‡	84 (73.7%)	48 (42.1%)	36 (31.6%)	< 0.001
McConnell's sign§	42 (38.5%)	8 (7.3%)	34 (31.2%)	< 0.001
Vital signs (paired data)				
Heart rate, beats/min||	97.9±18.9	86.5±14.0	11.4±19.4	< 0.001
Respiratory rate, breaths/min#	21.0±4.6	18.6±3.7	2.5±5.1	< 0.001
Patients on supplemental O_2_ **	144 (49.1%)	92 (31.4%)	52 (17.7%)	< 0.001
Supplemental O_2_ amount, L/min††,, ‡‡	2.3±5.1	1.0±2.5	1.3±5.2	< 0.001

Data are presented as mean±SD or n (%). The paired *t* test was performed on paired mean differences, and the McNemar test was performed on paired proportions.

TAPSE indicates tricuspid annular plane systolic excursion.

* n=92.

^†^ An increase in TAPSE after treatment from baseline.

^‡^ n=114.

^§^ n=109.

^||^ n=296.

^#^ n=282.

** n=293.

^††^ Patients who were not on supplemental O_2_ (ie, were on room air) were assigned an amount of 0 L/min for that visit.

^‡‡^ n=280.

Over the same follow‐up period, heart rate, respiratory rate, the number of patients on supplemental O_2_, and the supplemental O_2_ amount all decreased significantly (*P*<0.001; Table 5).

### Borg Dyspnea Scale

The median Borg dyspnea scale at rest decreased significantly from 4.0 [3.0–7.0] (somewhat severe) at baseline to 0.5 [0.0–1.0] (very, very slight [just noticeable]) at discharge, with a reduction of 3.5 [2.0–7.0] (*P*<0.001; Table 6).

**Table 6:** Changes of Borg Dyspnea Scale From Baseline to Discharge

	Baseline	Discharge	Reduction from baseline to discharge	*P* value
Borg dyspnea scale at rest (paired data)*	4.0 [3.0–7.0]	0.5 [0.0–1.0]	3.5 [2.0–7.0]	< 0.001

Data are presented as median [Q_1_–Q_3_]. The Wilcoxon signed rank test was performed on paired median differences.

* n=242.

### Secondary Safety End Points

The secondary safety end points occurred as follows: device‐related serious adverse events in 2 (0.7%) patients, all‐cause mortality within 30 days in 3 (1.0%) patients (2 [0.7%] from cardiovascular causes and 1 [0.3%] noncardiovascular death from progression of cancer diagnosed after the procedure), and symptomatic recurrent PE within 30 days in 2 (0.7%) patients (Table 7, Table S5).

**Table 7:** Secondary Safety End Points*

Device‐related serious adverse events†	2 (0.7%)
All‐cause mortality within 30 d	3 (1.0%)
Cardiovascular	2 (0.7%)
Noncardiovascular	1 (0.3%)
Symptomatic recurrent pulmonary embolism within 30 d	2 (0.7%)

Data are presented as n (%).

* Per independent medical reviewer.

^†^ Adverse events that were judged as probably or definitely related to the Indigo Aspiration System were considered to be device related.

## Discussion

This 300‐patient analysis is the largest cohort reported of patients treated with CAVT from the single‐arm, prospective, international, multicenter STRIKE‐PE cohort study. This analysis demonstrates efficiency, effectiveness, and safety of CAVT treatment of acute PE. In particular, the mean reduction of RV/LV ratio from baseline to 48 hours postprocedure was 26.8%, and the composite MAE rate at 48 hours postprocedure was 2.0%. The reduction of the right heart strain and anatomical changes occurred within 48 hours. Additionally, the median thrombectomy time was 30 minutes.

Excessive RV afterload is a key component of PE progression in intermediate‐risk and high‐risk patients with PE. Previous published data have demonstrated that EVT reduces the signs or symptoms of RV dysfunction, which may accompany hemodynamic instability in intermediate‐risk and high‐risk patients with PE.^11^, ^12^, ^14^, ^15^, ^21^, ^22^, ^23^, ^24^, ^25^, ^26^ One result from this interim analysis is an improvement in on‐table systolic pulmonary artery pressure immediately following thrombus removal within a short thrombectomy time; the improvement is comparable to those reported in other single‐arm prospective studies of EVT of PE.^11^, ^21^ Similarly, the improvement in RV/LV ratio (Table 8) in this analysis is consistent with the results from other studies of EVT of PE.^21^, ^22^, ^23^ Additional details on the right ventricle included improved TAPSE, RV diameter, and McConnell's sign, thus indicating anatomical changes that attenuate RV strain. Patients' heart rate, respiratory rate, and use and amount of supplemental O_2_ all improved in this analysis. Furthermore, perceived dyspnea improved by discharge. Altogether, these results suggest that CAVT could halt and reverse the progression of RV dysfunction to ameliorate hemodynamic status, heart rate, respiratory rate, oxygen use, and dyspnea. These insights on the effects of CAVT on the right ventricle can be used to facilitate decision‐making when treating this patient population with PE.

**Table 8:** Key Publications of RV/LV Ratio Changes Within 48 Hours After EVT of PE

Key studies	EVT device	PE mortality classification	Baseline RV/LV ratio	Follow‐up RV/LV ratio	Reduction in RV/LV ratio	*P* value
A Prospective, Multicenter Study of the Indigo Aspiration System Seeking to Evaluate the Long‐Term Safety and Outcomes of Treating Pulmonary Embolism analysis of 300 patients	Mechanical thrombectomy	Intermediate‐risk and high‐risk PE	1.40±0.36	0.99±0.22	0.40±0.30	< 0.001
FlowTriever All‐Comer Registry for Patient Safety and Hemodynamics, full US cohort results^21^	Mechanical thrombectomy	Intermediate‐risk and high‐risk PE	1.23±0.36	0.98±0.31	Not reported	< 0.0001
Ekosonic Registry of the Treatment and Clinical Outcomes of Patients With Pulmonary Embolism^23^	Catheter‐directed thrombolysis	Intermediate‐high and high‐risk PE	1.35±0.32	1.04±0.28	0.37±0.38	< 0.0001
Submassive and Massive Pulmonary Embolism Treatment With Ultrasound Accelerated Thrombolysis Therapy^22^	Catheter‐directed thrombolysis	Submassive and massive PE	1.55±0.39	1.13±0.2	0.42 ±0.36	< 0.0001

Data are presented as mean±SD. The paired *t* test was performed on paired mean differences.

EVT indicates endovascular treatment; PE, pulmonary embolism; and RV/LV, right ventricular/left ventricular.

Treatment decisions for patients with PE necessitate a careful consideration of both therapeutic benefits and potential adverse effects. Major bleeding is an established complication of systemic thrombolysis and could be as high as 9.9%, as reported in a meta‐analysis by Marti et al.^27^ In this analysis of 300 patients treated with CAVT, the composite MAE rate was 2.0% and was mainly due to major bleeding. This major bleeding rate is comparable to that reported in a published interim analysis by Toma et al. with a similar number of patients treated with a different mechanical thrombectomy device (1.2%).^13^ Additionally, in the current study, 3 of the 6 patients with major bleeding required blood transfusion, whereas in the study by Toma et al., all of the patients with major bleeding required transfusion.^13^ The major bleeding rate reported in the current study is also similar to that reported for a larger number of patients, in a meta‐analysis of treatment with mechanical thrombectomy (2.1%); the meta‐analysis did not report transfusion rates.^28^ When compared with other types of EVT devices, the major bleeding rate reported here is similar to that reported in a meta‐analysis for intermediate‐risk PE at 1.4% but lower than that reported for high‐risk PE at 6.7%.^29^ The low rates of MAEs, especially for major bleeding with transfusion, associated with CAVT make these devices a promising treatment option for intermediate‐risk and high‐risk PE.

Limitations of this study remain. This study is a single‐arm investigation and is thus not designed to directly compare CAVT with other PE treatments, such as anticoagulation, in a specific population with PE. Instead, this observational study provides information on the safety and effectiveness of CAVT on a spectrum of patients with PE, encompassing intermediate‐risk and high‐risk PE. This study also excluded patients with active cancer (stage III or IV cancer or cancer that requires active chemotherapy during the course of the study). This exclusion criterion precludes any direct conclusions regarding safety and effectiveness in that patient population. The device manufacturer's funding presents a potential source of bias; this is an additional limitation. To minimize bias, the following were instated: a steering committee, composed of external experts, was formed to guide study design, conduct, and analysis; an independent core laboratory reviewed imaging data including the RV/LV ratio for most patients (71.6% for this interim analysis; Table S4); an independent medical reviewer adjudicated all end point events; and a diverse group of investigators and sites was recruited to reduce any single site's or region's influence on study outcomes. These limitations do not take away from this study's strengths, which include its prospective design, international participation, large sample size, 1‐year follow‐up, and multiple outcome measures.

### Conclusions

In conclusion, this interim analysis of STRIKE‐PE shows that treatment of intermediate‐risk and high‐risk patients with PE with CAVT devices is rapid and safe and was associated with significant improvements in RV/LV ratio, other periprocedural outcomes, and dyspnea. Whereas the results in this interim analysis highlight important periprocedural outcomes, STRIKE‐PE's ultimate goal of reporting patient‐centric outcomes at 90 days and 1 year will increase understanding of recovery after CAVT treatment of acute PE and help inform the management of complex PE.

## Sources of Funding

This study was sponsored by Penumbra, Inc.

## Disclosures

John M. Moriarty reports consulting fees from AngioDynamics, Innova Vascular, Inquis Medical, Boston Scientific, Imperative Care, and Thrombolex; payment or honoraria for lectures, presentations, speakers bureaus, or educational events from AngioDynamics and Penumbra; and stock or stock options from Pavmed and Thrombolex. Brian J. Schiro reports payment or honoraria for lectures, presentations, speakers bureaus, educational events from Penumbra (personal payment for speaking), Cook Medical, Philips, and Medtronic. Suhail Y. Dohad reports grants or contracts from any entity if not for the present article from Penumbra (research grant) and consulting fees from Penumbra. Hugh C. Davis reports consulting fees from Penumbra and ProKidney. David M. Shavelle reports grants or contracts from any entity if not for the present article from Penumbra (research support), Neurescue (research support), and V‐Wave Medical (research support). George S. Chrysant reports consulting fees from Abbott Vascular, Boston Scientific, Medtronic, Penumbra, Philips, and Shockwave and participation on an advisory board for Abbott Vascular and Medtronic. Elias A. Iliadis reports payment or honoraria for lectures, presentations, speakers bureaus, educational events from Penumbra (speakers bureau) and Janssen (speakers bureau). David J. Dexter reports consulting fees from Boston Scientific, Penumbra, Inari Medical, and AngioDynamics. Frances M. West reports consulting fees from the steering committee for STRIKE‐PE sponsored by Penumbra (individual); payment for expert testimony from Spear, Greenfield, Richman, Weitz & Taggart, PC (individual) and from German, Gallagher & Murtagh, PC (individual); leadership or fiduciary role in other board, society, committee or advocacy group, paid or unpaid from the PERT Consortium Board of Directors. Brent Keeling reports consulting fees from AngioDynamics (individual), Penumbra (individual), and Dexcom (individual) and stock or stock options from Viz AI (individual). Andrew S. P. Sharp reports consulting fees from AngioDynamics, Boston Scientific, Philips, Penumbra, Medtronic, and Recor Medical; payment or honoraria for lectures, presentations, speakers bureaus, or educational events from AngioDynamics, Boston Scientific, Philips, Penumbra, Medtronic, and Recor Medical; support for attending meetings or travel from Boston Scientific, Philips, Penumbra, Medtronic, and Recor Medical; and stock or stock options from Althea Medical. Ido Weinberg reports consulting fees from Penumbra, Magneto Thrombectomy, and Daiichi Sankyo. The remaining authors have no disclosures to report.

## Supporting information

Tables S1–S5
