# Supplementary material for: Periprocedural Results and Right Ventricular Outcomes of Computer Assisted Vacuum Thrombectomy Treatment of Acute Pulmonary Embolism: Interim Analysis of 300 Patients From the STRIKE‐PE Study
Source: J Am Heart Assoc. 2025 Aug 29;14(17):e039975. doi: 10.1161/JAHA.124.039975 (PMC12553419; doi:10.1161/JAHA.124.039975)
Supplement: Supplementary file 1 — Tables S1–S5 [file JAH3-14-e039975-s001.pdf]

SUPPLEMENTAL MATERIAL

Table S1. Additional site principal investigators and treating physicians of this STRIKE-PE analysis

Listed in alphabetical order
Chaitanya Ahuja, Aleksander Araszkiewicz, Massoud Allahyari, Hamed Aryafar, Antonious S. Attallah, Iftekhar Baig, Rohit Bhatheja, B. Kirke Bieneman, Vivian L. Bishay, Ankur Chawla, Kiran V. Chunduri, Bryan D. Cogar, Mircea M. Cristescu, Donna L. D'Souza, Tim S. Daly, Amir Darki, Hosem F. El Sayed, Fakhir F. Elmasri, Anthony Feghali, H. Gabriel Lipshutz, Ripal T. Gandhi, Kirema I. Garcia-Reyes, Brian S. Geller, Todd W. Gensler, Marat Goldenberg, Jafar Golzarian, Jose Andres Guirola Ortiz, Mustafa Haddad, Robert E. Heithaus, Khiet C. Hoang, Mohamed I. Kabley, Hani Kador, Martyn Knowles, Grzegorz Kopeć, Aaron D. J. Kulwicki, Vladimir Lakhter, Michael P. Lazarowicz, Daniel E. Long, Robert A. Lookstein, Santhosh J. Mathews, Ari J. Mintz, Patrick E. Muck, Sílvio Renato Narciso Leal, Andrew S. Niekamp, Siddharth A. Padia, Eugene Paik, Jean M. Panneton, Pedro Pardo Moreno, Jong H. Park, Constantino N. Pena, Paul E. Perkowski, Darren W. Postoak, Alex Powell, Aditya S. Prasad, Trent L. Proffitt, Mona Ranade, Animesh Rathore, Kaili C. Redifer Tremblay, Michael S. Rosenberg, Jeffrey E. Rossi, Gonzole Ruiz Verde, Rahul Sakhuja, Robert M. Schainfeld, Matthew J. Scheidt, Jessica L. Secor, Sanjum S. Sethi, Muhammad U. Shahid, Dan Shilo, Sylwia Sławek-Szmyt, Peter A. Soukas, Jeffrey M. Sparling, Ravi N. Srinivasa, Jonathan D. Steinberger, Jakub Stępniewski, Lindsay K. Thornton, Jeffrey D. Vogel, Lawrence R. Whitney III, and Joe D. Zachary. The authors also acknowledge the Cardiovascular Research Foundation as the core lab for evaluating the echocardiographic images.

Table S2. Laboratory values at presentation

	Value (N = 300)
Hemoglobin, g/dL	13.4 ± 2.0
Hematocrit, %	40.5 ± 5.5
Platelet count, 10^3 cells/μL	215.1 ± 82.4
BNP, pg/mL	148.0 [60.0-431.0][†]
NT-proBNP, pg/mL	1498.0 [542.0-3728.0][‡]
Troponin I, ng/mL	0.2 [0.0-0.5][§]
Troponin T, ng/mL	0.1 [0.0-0.1][‖]
International normalized ratio	1.1 ± 0.1[#]
Prothrombin time, s	13.5 ± 2.0[**]
Activated partial thromboplastin time, s	30.3 [27.0-47.0][††]
Partial thromboplastin time, s	29.7 [27.0-35.7][‡‡]
D-dimer, ng/mL	8120.0 [3750.0-18240.0][§§]

Data are presented as mean ± SD or median [Q_1-Q_3].
BNP, brain-type natriuretic peptide; NT-proBNP, N-terminal pro–brain natriuretic peptide.
[†]n = 115.
[‡]n = 163.
[§]n = 183.
[‖]n = 116.
[#]n = 259.
[**]n = 252.
[††]n = 158.
[‡‡]n = 124.
[§§]n = 143.

Table S3. Technical periprocedural characteristics

	Value (N = 300)
Patient sedated during procedure	249 (83.0%)
Highest level of sedation	
Conscious sedation	236 (94.8%)[*]
General anesthesia	13 (5.2%)[*]
Access site	
Left femoral vein	14 (4.7%)
Right femoral vein	283 (94.3%)
Right internal jugular vein	3 (1.0%)
Treatment locations (multiple treatment locations allowed)	
Main pulmonary artery	45 (15.0%)
Bilateral	244 (81.3%)
Right pulmonary artery	125 (41.7%)
Right upper lobe	219 (73.0%)
Right interlobar segment	255 (85.0%)
Right middle lobe	227 (75.7%)
Right lower lobe	203 (67.7%)
Left pulmonary artery	117 (39.0%)
Left upper lobe	142 (47.3%)
Left interlobar segment	183 (61.0%)
Lingula	181 (60.3%)
Left lower lobe	213 (71.0%)
Indigo Aspiration System accessed pulmonary embolus	300 (100%)
Indigo devices used	
Aspiration catheters	
Lightning 12	205 (68.3%)
Lightning 12 ×2	4 (1.3%)
Lightning 12 + CAT8	4 (1.3%)
Lightning 12 + Lightning 7	1 (0.3%)
Lightning Flash	83 (27.7%)
Lightning Flash ×2	2 (0.7%)
Lightning Flash + Lightning 12	1 (0.3%)
Number of separators used	
0	136 (45.3%)
1 (Separator 12)	164 (54.7%)
Adjunctive treatment used	13 (4.3%)
Within 24 h before procedure	5 (1.7%)[†]
During thrombectomy	1 (0.3%)[‡]
After thrombectomy and before venous access site closure	7 (2.3%)[§]

Data are presented as n (%).
[*]n = 249.

[†]Intravenous tissue plasminogen activator (tPA), 5.

[‡]Intra-arterial tPA, 1.

[§]Intra-arterial tPA, 4; ultrasound-assisted catheter-directed thrombectomy (UACDT), 2; intra-arterial tPA and UACDT, 1.

Table S4. Imaging modality pair classifications for measuring right ventricular/left ventricular ratio

	**Value (N = 300)**
Matched images available for measuring RV/LV ratio	285 (95.0%)
CTPA, evaluated by core lab	122 (42.8%)[*]
Echocardiography, evaluated by core lab	82 (28.8%)[*]
CTPA, evaluated by physician	56 (19.6%)[*]
Echocardiography, evaluated by physician	25 (8.8%)[*]
Matched images unavailable for measuring RV/LV ratio	15 (5.0%)

Data are presented as n (%).
CTPA, computed tomography pulmonary angiography; RV/LV, right ventricular/left ventricular.
[*]n = 285.

Table S5. Additional details of primary and secondary safety endpoint events

Safety endpoints	Additional details
Primary safety composite major adverse events	Major bleeding alone (4 patients)<br>– Access site hematoma related to the index procedure.
	Major bleeding, device-related pulmonary vascular injury, and device-related clinical deterioration (1 patient)<br>– Pulmonary artery perforation.<br>– Considered a device-related serious adverse event (SAE).<br>– Patient fully recovered.
	Major bleeding and device-related clinical deterioration (1 patient)<br>– Pulmonary artery hemorrhage.<br>– Considered a device-related SAE.<br>– Patient was stabilized with extracorporeal membrane oxygenation and on postprocedure day 2 underwent a second procedure involving an alternative aspiration thrombectomy device and administration of 10 mg of intra-arterial tissue plasminogen activator. Patient continued to have a complicated hospital course, resulting in death from endocarditis on postprocedure day 21.
Secondary safety endpoints	Device-related SAEs (2 patients)<br>– Patient 1: Pulmonary artery perforation (see the second box of “Primary safety composite major adverse events” above for more information).<br>– Patient 2: Pulmonary artery hemorrhage (see the third box of “Primary safety composite major adverse events” above for more information).
	All-cause mortality within 30 days (3 patients)<br>– Patient 1: Patient died, on postprocedure day 21, of endocarditis (cardiovascular cause of death; see the third box of “Primary safety composite major adverse events” above for more information).<br>– Patient 2: Patient had an uneventful thrombectomy procedure but suffered from multiple comorbidities including chronic heart failure and an active COVID-19 infection. Patient died, on postprocedure day 2, of right ventricular failure attributed to recurrent PE (cardiovascular cause of death).<br>– Patient 3: Patient died, on postprocedure day 15, from progression of adenocarcinoma (noncardiovascular cause of death) that was diagnosed after the procedure.
	Symptomatic recurrent pulmonary embolism (PE) within 30 days (2 patients)<br>– Patient 1: Patient continued to experience PE symptoms with a repeat computed tomography pulmonary angiography revealing unchanged thrombus burden from baseline imaging and was treated with a second thrombectomy procedure. Patient fully recovered.<br>– Patient 2: Patient died, on postprocedure day 2, of right ventricular failure attributed to recurrent PE (see “Patient 2” under “All-cause mortality with 30 days” above for more information).
